# An evaluation of China’s new rural cooperative medical system: achievements and inadequacies from policy goals

**DOI:** 10.1186/s12889-015-2410-1

**Published:** 2015-10-23

**Authors:** Chengyue Li, Yilin Hou, Mei Sun, Jun Lu, Ying Wang, Xiaohong Li, Fengshui Chang, Mo Hao

**Affiliations:** Research Institute of Health Development Strategies, Fudan University, 130 Dong An Road, Shanghai, 200032 China; Collaborative Innovation Center of Social Risks Governance in Health, 130 Dong An Road, Shanghai, 200032 China; Department of Public Administration and International Affairs, Maxwell School of Citizenship and Public Affairs, Syracuse University, 426 Eggers Hall, Syracuse, NY 13244-1020 USA

**Keywords:** China, New rural cooperative medical system (NRCMS), Policy goals, Implementation, Evaluation

## Abstract

**Background:**

Although much public scrutiny and academic attention has focused on the evaluations of system implementation since the beginning of New Rural Cooperative Medical System (NRCMS) in China, few studies have systematically evaluated the achievements of the stated policy goals. The purpose of this study is to examine to what extent the policy goals of NRCMS have been achieved.

**Methods:**

Using multistage sampling processes, two rounds of cross-sectional household surveys including 9787 and 7921 rural households were conducted in Eastern China in year 2000 and year 2008, respectively. A pre- and post-implementation comparison was used to evaluate the achievement of policy goals in three measures: impoverishment from major health hazards, household financial risk from medical expenses, and rural income inequity. Intention surveys were also applied to find out potential obstacles in the implementation of NRCMS.

**Results:**

The rate of re-impoverishment from health hazard was reduced from 2.69 % ex ante to 2.12 % ex post, a decrease of 21.13 %. The severity of impoverishment fell from a previous 4.66 % to 3.02 %, a decline of 35.18 %. Economic risk of medical treatment population relative to the whole population fell from 2.62 ex ante to 2.03 ex post, a 22.52 % reduction. As indication of effect on improving income equity, the Gini coefficient fell from 0.4629 to 0.4541. The effects of NRCMS were significantly better than those of RCMS. Despite the preliminary achievements, our intention survey of key respondents identified that technical difficulties in actuarial funding and more sustainable reimbursement schedules has become the most challenging barriers in achieving the goals of NRCMS, while raising the insurance premium on NRCMS was no longer as big a barrier.

**Conclusions:**

With NRCMS, China has established a medical security system to reduce the financial burden of healthcare on rural residents. NRCMS has achieved some positive though limited effects; but technical difficulties in the implementation of NRCMS have become barriers to achieve the pre-set policy goals. Efforts should be made to improve the capacity building in the design of the reimbursement schemes for the implementers of NRCMS, such as identifying medical impoverishment, calculating actuarial funding levels for the risk pooling.

## Background

The extent of rural development is an important indicator of the country’s economic prosperity in China; it is also the basis for social stability and progress of the whole society, especially for developing countries that have a large rural population and drastic rural–urban income disparity [1]. In China, about 70 % of the population, nearly 900 million, were classified as rural residents (by the still existing “household registration” system) [2]. Relative to urban dwellers, these rural residents are the largest low-income group. They do not have the privilege of health insurance coverage as most urban dwellers, which exacerbates the long-existing, already drastic rural–urban income disparity and implants underlying threats to long-run social stability. Therefore, providing health insurance coverage for rural populations is increasingly regarded as not only an important health protection measure, but also an important part of rural development.

In fact, China’s serious effort in providing health care for its rural population could date back to the 1950′s when the country was still very poor [3]. The Chinese government experimented with what was later known as the “Rural Cooperative Medical System” (RCMS) as the major means to provide basic healthcare and to prevent epidemic diseases [4]. The word “cooperative” holds the key: rural residents were placed into units of collective economy, by which everyone household contributed to a revenue pool and was provided a basic level of healthcare protection that was funded by the pool. By the late 1970s, it covered 90 % of the rural population [5, 6]. However, with the disappearance of the collective economy during China’s market-oriented economic reform that started in the 1980s, the RCMS collapsed by the 1990s [7], except in the more urban economically developing provinces in Eastern China. This left nearly 80 % of the rural population did not have any health coverage [8]. Since then, medical expenses sky-rocketed along with high-rate economic growth, which outstripped the growth of personal income [9, 10]. The rural residents were confronted with unaffordable health care and for those who were unfortunately chronically or terminally ill, impoverishment due to medical expenses became a vicious circle between illnesses and poverty [11]. Unaffordable health services and poverty due to medical expenses became daily troubles to low-income rural residents, the couplet of which created a serious threat to the overall social stability and economic progress of the nation.

Against this background, the Chinese government began to readdress rural health care and launch the policy of “New Rural Cooperative Medical System” (NRCMS) in 2003 [12]. According to the 2003 official guideline, NRCMS is defined as a system of mutual assistance for health protection through risk-pooling. The system is guided, organized and subsidized by the central, provincial, and county governments. It incorporates two important policy features: voluntary participation by the rural population and emphasis on protection against catastrophic illnesses. Its policy goals are explicitly designed to mitigate the financial burden of healthcare on rural residents, to minimize poverty caused by large medical service bills, and to promote (after medical-expenses) income equity. Last but not least, the system is to balance its revenue and outlay in order to maintain sustainability of the insurance fund. It has been calculated that by the end of 2013, 98.7 % (802 million) of the rural population would be covered by the NRCMS, which was the world’s largest health insurance program [13].

Since the beginning of NRCMS, much public scrutiny and academic attention has focused on the evaluations of system implementation such as coverage rate, enrollee satisfaction, ratio of reimbursement, deductible, fund management and supervision, health care utilization, and medical expenses [14–17]. Several studies have also attempted to evaluate the effects of NRCMS on alleviating health-related poverty or financial protection [18–21]. However, few studies have systematically evaluated the achievements of the stated policy goals. In other words, while the expansion of NRCMS coverage since 2003 is impressive, it is not clear whether and to what extent the policy goals of NRCMS have been achieved, and whether the effects of NRCMS are better than those of RCMS. Thus, it is of great significance to scrutinize the implementation of the program with an evaluation of the achievement of the policy goals. The results will also be of valuable to the sustainable development of NRCMS and to help to refine the implementation of the program.

The purpose of this study is to offer an evaluation of to what extent the policy goals of NRCMS have been achieved. Specifically, we take pre-reimbursement of medical expenses by program participants as ex ante and post-reimbursement as ex post, and before and after the implementation of NRCMS as contrast, to examine the actual changes in three specific measures (as proxy of the stated policy goals): impoverishment from major health hazards, financial risk to patients from medical expenses, and rural income (in) equity. In addition, we also explored potential difficulties in implementing NRCMS so as to propose strategies to help NRCMS achieve its policy goals in the future.

## Methods

### Study design and sampling

Two rounds of cross-sectional surveys were conducted in year 2000 and year 2008, respectively. Each survey contained two sections. Household survey was used to analyze the utilization of medical services and financial burden from out-of-pocket medical expenses under (N)RCMS. Intention survey was used to find out potential difficulties or obstacles in implementing the healthcare policy and achieving the policy goals.

The study was conducted in three cities, Jiading in Shanghai municipality, Changzhou in Jiangsu province, and Weifang in Shandong province, which are all located in the relatively well-developed Eastern Region of China. In 2008, the national per capita GDP was 22,698 RMB (1 RMB was about 0.144 U.S. dollar in 2008) in the mainland that year. Shanghai, Jiangsu and Shandong were ranked the 1st (72,553 RMB), 5th (39,484 RMB) and 9th (32,995 RMB) on per capital GDP, respectively [22]. Jiading district was ranked the fifth in terms of per capita GDP among the 9 suburban districts in Shanghai, with a GDP per capita of 62,100 RMB [23]. Changzhou city’s per capita GDP was 61,504 RMB, and it was ranked the fourth among the 13 cities in Jiangsu province in per capita GDP [24]. Weifang city was ranked the ninth in terms of per capita GDP among the 17 cities in Shandong province and its per capita GDP was 28,106 RMB in 2008 [25].

The household survey used multi-stage, stratified, random-group sampling processes [26]: First, the sampled areas were divided into three groups based on their level of economic development; three townships were randomly selected from each locality in each economic group; then, three villages were randomly chosen from each sampled township; and finally 20 % of households were randomly selected in each sampled village. A total of 9787 rural households were included in the first round survey (year 2000); the size in the second round (year 2008) was 7921 households.

Along with the two rounds of household surveys, qualitative method such as intention survey was carried out with policy makers (government officers from local government, health department, and financial department), NRCMS and health facility managers, and health providers and physicians from different levels of health facilities at the county, township and village levels by structured questionnaires. We explored their perceptions on ten potential difficulties (listed in Table 4) in, or obstacles to, implementing the rural healthcare system and achieving the design purposes, and examined whether the ranking of the potential difficulties have changed before (year 2000) and after (year 2008) the implementation of NRCMS. In total, 2298 persons in year 2000 and 6170 persons in year 2008 who have completed the questionnaires were included in the intention survey.

Approval (IRB#08–03–0130) for the study was gained from the Medical Research Ethics Committee at the School of Public Health of Fudan University in China (IRB00002408&FWA00002399). All respondents in the survey have read a statement that explained the purpose of the survey and gave consent to continue.

### Data collection

The household surveys were conducted in July to September in 2000 and July to October in 2008, respectively. After receiving training on the contents of the questionnaire and interview techniques, teachers, graduate students and undergraduate students from Fudan University and Weifang Medical College, and local Center for Disease Control and Prevention (CDC) staff acted as the interviewers. A structured questionnaire was used to conduct face-to-face interviews with household members about general demographic information of households (age, gender, annual household income and expenditure, and health insurance status), the rate and frequency of morbidity in a two-week period, healthcare needs, utilization of medical services, and burden of medical expenses including outpatient and hospital expenses in the previous 12 months.

The intention surveys were carried out with the support of the local Health Bureau in Jiading, Changzhou and Weifang. The structured questionnaires including the opinions on the barriers to fulfill the policy of (N)RCMS were completed by the selected NRCMS officials, government officials and medical staff, and sent back to the research team.

Completed questionnaires from household surveys and intention surveys were checked by research team members. Any missing questions and errors were corrected with the interviewers, or by connecting to the household or respondents when necessary. To test the representativeness of our data, we constructed the Myer’s Index from our year-2000 survey data against data from China’s Year 2000 Census and our 2008 survey data against China’s Fourth National Health Services Survey (2008) in terms of age structure [27]. A Myer’s index larger than 60 indicates statistically significant difference in age structure between the two samples compared. Our constructed Myer’s index was 6.46 for the year-2000 survey and 7.97 for the year-2008 survey; that is, our data well represented the national population for age and population structure and we have a reliable data basis for empirical analysis.

### Measures of policy goal achievement

We developed three measures as proxy to the three policy goals in the analysis in evaluating the achievement of the policy goals. The three measures are: medical impoverishment, financial risk to households from medical expenses, and income inequity after the payment of medical bills. All three measures are calculated on the basis of household which is the socio-economic unit to share large medical expenses and for rural residents to join the NRCMS.

**Medical impoverishment** (or poverty from major health hazards) refers to incidences where the medical expenses from a major illness exceed a household’s ability-to-pay so that the after-bills-payment family income falls below the poverty line. In this study, the poverty line is defined as half of the average household income in a region as has been used in the public health literature [28]. A household’s ability-to-pay is taken as the difference between the average household income and the poverty line [29].

We measure medical impoverishment by its rate and severity [30]. The rate of impoverishment from health hazards reflects the overall level of poverty; it is expressed as:

1 $$ U\left(\%\right)=\frac{P}{N}\times 100\% $$

where *U* indicates the rate of medical impoverishment; *P* is the number of households that have been impoverished by medical expenses; and *N* is the total number of households.

The severity of medical impoverishment is the ratio of total amount of medical expenses exceeding total ability-to-pay of households in poverty over the total amount of ability-to-pay for households of the entire population. The formula is:

2 $$ S\left(\%\right)=\frac{{\displaystyle {\sum}_{i=1}^P\left({M}_i-R\right)}}{{\displaystyle {\sum}_{i=1}^NR}}\times 100\% $$

where *S* is the (rate of) severity of medical impoverishment; *M*_*i*_ is the *i-*th household’s medical expenses; *R* is the household’s ability-to-pay (i.e., revenue or income); and *N* is the total number of households.

**Financial risk from medical expenses** refers to financial losses patients and their families bear from illness. It is defined as the probability of residents in a specified area paying an amount of medical expenses in a period, and the financial losses under this probability [31]. We use an index of “relative risk” in epidemiology to estimate this probability [32] – it is the multiple of the relative risks of a specific medical treatment group to the entire population [33]. The formula is:

3 $$ RR=\frac{\left({\sum}_{i=1}^n{E}_i\right)/n}{\left({\sum}_{i=1}^N,,{E}_i\right)/N} $$

where *RR* is relative risk from medical expenses; *E*_*i*_ is *i-*th person’s total annual medical expense; *n* is size of the specific medical treatment group; and *N* is the population.

**Fig. 1:**
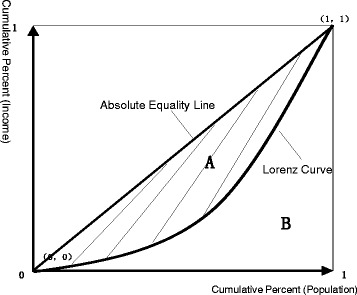
Sketch Map of Lorenz Curve

We use the Gini coefficient to evaluate income equity (among rural residents) – whether reimbursement from the NRCMS on eligible medical expenses can partly reduce the impact of healthcare burden on household income and thereby improve income equity. The Gini coefficient is a judgment index of income distribution [34]. As illustrated in Fig. 1, the area between the Lorenz Curve and the absolute equality line is *S*_*A*;_ the area at the lower right below the Lorenz Curve is *S*_*B*_. The Gini or Lorenz Coefficient is defined as the quotient of *S*_*A*_ divided by the sum of *S*_*A*_ and *S*_*B*_, in a range between 0 and 1. Smaller coefficients indicate more equitable income distribution [35]. The formula is as follows [36]:

4 $$ G=\frac{S_A}{S_A+{S}_B}=\frac{S_A}{0.5}=2{S}_A=1-2{S}_B $$

### Data analysis

Household survey data and intention survey data were all entered into a database using Excel 2010, and were analyzed using SPSS 13.0.

**Fig. 2:**
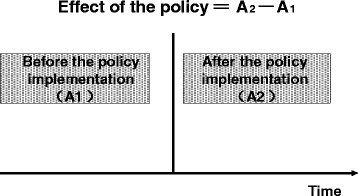
Before-and-after Comparison Analysis of Policy Effect

A pre- and post-implementation comparison was used to evaluate the achievement of policy goals – how the outcomes change over time as policies are implemented. As illustrated in Fig. 2, *A*_*1*_ stands for the outcome as ex ante and *A*_*2*_ for the outcome as ex post; the difference between *A*_*2*_ and *A*_*1*_ (*A*_*2*_*- A*_*1*_) represents the effect of implementing the policy [37]. Thus we can figure out to what extent impoverishment from major health hazards has been alleviated, financial risk from healthcare expenses has been reduced, and income equity that has been eroded by medical expenses has now been improved through reimbursement of eligible medical expenses. The calculation formula is as follows:

5 $$ Effect\left(\%\right)=\frac{I_{ex- post}-{I}_{ex- ante}}{I_{ex- ante}}\times 100\% $$

where *Effect* stands for effect of the policy; *I*_*ex ante*_ is value of the effect evaluation index before reimbursement; *I*_*ex post*_ is value of the effect evaluation index after reimbursement; and the effect ratio indicates change of/in the effect evaluation index before and after reimbursement.

Statistical analyses (T tests or chi-squared tests) were performed to examine potential differences in indexes (medical impoverishment and financial risk from medical expenses) between pre- and post-reimbursement, and the reductions before and after the implementation of NRCMS. Dominance test was carried out to ascertain whether the reimbursement reduced the income inequity [36].

## Results

### Important parameters of the two surveys

Important characteristics of rural residents who use the new medical system that are relevant to this study include the following. The average annual household income increased by about 66 % from 18,307 RMB in 2000 to 30,501 RMB in 2008. In the latter year, 92.7 % of households participated in NRCMS, whereas only 73.6 % participated in RCMS in year 2000 (As we said in the Background section, the sampled areas had strong local collective economy so that the old RCMS was never completed disrupted). Between the two survey years, however, medical expenses increased exponentially: The outpatient costs per doctor’s visit went up by over five-fold, from 225 RMB to 1325 RMB and the average hospital-stay costs more than doubled, from 3138 RMB to 7342 RMB. The average reimbursement ratio, in the year-2000 RCMS and the year-2008 NRCMS, rose for both outpatient expenses (11.7 % in 2000 vs. 19.1 % in 2008) and inpatient expenses (18.0 % in 2000 vs. 21.2 % in 2008). Details are shown in Table 1.

**Table 1:** Characteristics of households in healthcare utilization

Variable	Year 2000	Year 2008
Household surveyed	9787	7921
Average annual household income (RMB)	18,307	30,501
Coverage of healthcare among rural residents (%)	73.6	92.7
Outpatient costs per visit (RMB)	225.0	1325.0
Average outpatient reimbursement ratio (%)	11.7	19.1
Average inpatient costs (RMB)	3138.0	7342.0
Average inpatient reimbursement ratio (%)	18.0	21.2

### Achievement of NRCMS policy goals

Achievement of the three major goals of NRCMS is shown in Tables 2 and 3. In terms of mitigating medical impoverishment from health hazards, the rate has been reduced from 2.69 % ex ante to 2.12 % ex post, which is a decrease of over 21 % (*p* <0.05). In 2000, this rate had dropped from 2.61 % ex ante to 2.16 % ex post, a decrease of 17.25 % (*p* <0.05). The severity of medical impoverishment fell from 4.66 % ex ante to 3.02 % ex post, a decline of 35.18 % (*p* <0.01), which was higher than the 22.84 % drop in 2000. As to reducing financial risk from medical expenses, the risk of medical treatment population relative to the whole population fell from 2.62 ex ante to 2.03 ex post, a 22.52 % reduction in 2008 (*p* <0.01) which was higher than it was in 2000 (from 1.25 ex ante to 1.09 ex post, a 12.81 % reduction). Finally, as effect on improving income equity eroded with medical expenses, the Gini coefficient among rural residents in 2008 increased from 0.4292 to 0.4629 with payment of medical expenses and then fell to 0.4541 after NRCMS reimbursement, a reduction of 26 %. The improvement with the RCMS in 2000 was also obvious at 12.45 % (increase by 0.0273 with payment of medical expenses and reduction of 0.0034 after RCMS reimbursement). Dominance tests showed that the Lorenz curves for household income after reimbursement dominated the Lorenz curves before reimbursement both in 2000 and 2008, suggesting that the compensation from RCMS and NRCMS both improved the income equity. Furthermore, the new program (NRCMS) has shown significantly better results than the old one (RCMS) on all three measures.

**Table 2:** Achievements of policy goals – RCMS (2000) vs. NRCMS (2008)

Variable	RCMS (2000)			NRCMS (2008)		
	A	B	C	D	E	F
	Ex ante^a^	Ex post^b^	% Change^c^	Ex ante	Ex post	% Change
Impoverishment from health hazards (%)	2.61	2.16	−17.25^d^	2.69	2.12	−21.13^d,e^
Severity of impoverishment (%)	4.89	3.78	−22.84^d^	4.66	3.02	−35.18^d,e^
Medical financial risk	1.25	1.09	−12.81^d^	2.62	2.03	−22.52^d,e^

^a^Ex ante = after payment of medical bills

^b^Ex post = after reimbursement of medical bills from RCMS or NRCMS

^c^Percent change (%∆) = (B – A)/A*100 or (E – D)/D*100

^d^Significant difference in changes between pre- and post-reimbursement

^e^Significant difference in changes between RCMS and NRCMS

**Table 3:** Achievement of policy goals – RCMS (2000) vs. NRCMS (2008)

Variable	RCMS (2000)				NRCMS (2008)			
	A	B	C	D	E	F	G	H
	Initial^a^	Ex ante^b^	Ex post^c^	% Change^d^	Initial	Ex ante	Ex post	% Change
Income equity^e^	0.4126	0.4399	0.4365	−12.45	0.4292	0.4629	0.4541	−26.11^f^
Dominance test^g^	–	D-	D+	–	–	D-	D+	–

^a^Initial = before payment of medical bills

^b^Ex ante = after payment of medical bills

^c^Ex post = after reimbursement of medical bills from RCMS or NRCMS

^d^Percent change (%∆) = (C – B)/(B – A)*100 for RCMS and (G – F)/(F – E) *100 for NRCMS

^e^Income equity = Gini coefficient

^f^Significant difference in changes between RCMS and NRCMS

^g^“D-” indicated that the Lorenz curves after payment of medical bills (B in year 2000; F in year 2008) was dominated by the Lorenz curves before payment of medical bills (A in year 2000; E in year 2008). “D+” indicated that the Lorenz curves after reimbursement of medical bills (C in year 2000; G in year 2008) dominated the Lorenz curves before reimbursement (B in year 2000; F in year 2008)

### Potential difficulties in implementing NRCMS

As mentioned in section 2, obstacles were identified from intention surveys of key respondents as ten potential difficulties in implementing NRCMS. Solving these obstacles will help achieve the design goals of the new medical system. As displayed in Table 4, “technical difficulties in policy implementation” was ranked the sixth in 2000 but became the top obstacle in 2008; whereas “inadequate government attention and insufficient policy authority,” which was ranked the first obstacle in 2000, dropped to the second place in 2008. The issue of “necessary funding level unclear” retains the same rank order in 2008 as in 2000. In addition, “township and village finance too weak to meet the matching requirement of (N)RCMS” was ranked second in 2000 but fell to the sixth in 2008, and “rural residents did not have enough confidence in NRCMS” dropped from the third to the ninth spot over the same period. With the promotion of NRCMS and the diversification of funding sources, “inadequate government attention” and “weak local finance” were no longer major obstacles to achieving the policy goals. “Technical difficulties of actuarial funding” and “more sustainable, practicable reimbursement schedules” became the most challenging barriers.

**Table 4:** Ten obstacles in implementing RCMS (2000) and NRCMS (2008)

#	Obstacles to implementation	Constituent ratio (%)		Rank order	
		2000	2008	2000	2008
1	Insufficient government attention; inadequate policy authority	24.1	15.1	1	2
2	Responsibilities not clear between relevant departments	6.9	7.8	8	8
3	Multiple technical difficulties in policy implementation	8.2	16.2	6	1
4	Township and village finance inadequate to fund (N)RCMS	11.0	9.5	2	6
5	Policy not obligatory; implementation at discretion of local officials	8.0	9.7	7	5
6	Support for (N)RCMS varies among local government, healthcare organizations, village doctors, and rural residents	6.1	7.8	9	7
7	Lack of coordination between government departments to achieve policy goals	5.8	2.8	10	10
8	Needed funding level not clear	10.4	13.5	3	3
9	Rural residents lack confidence in (N)RCMS	10.4	6.0	3	9
10	(N)RCMS does not target most urgent demands of rural residents	9.1	11.6	5	4
Total		100.0	100.0	–	–

## Discussion

Our findings show that NRCMS has achieved some positive effects on the goals of reducing the financial burden of healthcare, mitigating impoverishment from health hazards, and improving income equity eroded by medical expenses, which are similar to and confirm those from other studies. Yip and Hsiao [18] showed that NRCMS could reduce the poverty headcount by 3.5–3.9 % and the average poverty gap by 11.8–16.4 %. Sun et al. [19] found NRCMS generated a 20.4 % decrease in medical impoverishment and a 19.2 % decline in the average poverty gap in rural areas of Shandong Province. Over the past few years of implementation, the funding level of NRCMS has steadily increased, with per capita annual government subsidy increasing from 40 RMB in 2003 to 120 RMB in 2010. Meanwhile, inpatient reimbursement ratio has been raised, with its cap raised to more than eight times the national net income of rural residents [38]. In sum, adequate funding with large risk-pools and higher reimbursement ratio are major factors leading to the success of NRCMS so far.

Our findings also indicated that there was greater reduction in impoverishment, medical financial risk and income inequality through reimbursements under NRCMS than RCMS. It is probably due to several important features of NRCMS in comparison to its predecessor, the RCMS. First, NRCMS is co-financed by the central government, provincial and local governments, and individuals; thus the funding sources are more diversified, stable, and reliable. In contrast, RCMS was financed mainly with individual payments plus some township and village input, with no central, provincial and county government subsidies; thus its funding sources were neither stable nor reliable. Second, operating at the county level rather than the township or village level, NRCMS possesses a much larger pool to dilute and share financial risks. Third, policies and guidelines are promulgated by the central government, and relevant agencies are established in the central and local governments to manage and supervise the implementation of NRCMS. In contrast, RCMS was managed mostly by village doctors or rural residents themselves, with no effective guidance or supervision. Finally, NRCMS gives priority to catastrophic illnesses, the most urgent demand of the rural population; voluntary participation with the household as the unit makes it more acceptable. These are the reasons why NRCMS has advanced beyond RCMS [39, 40].

Despite the above mentioned successes, the outcome of NRCMS has not yet met all the expectations, with only 30 % reduction of medical impoverishment and little effect on improving income equity. China’s NRCMS still has a long way to go in obtaining the policy goal of reducing, ultimately eliminating impoverishment from health hazard in rural areas. There are several explanations for this limited outcome. First, the focus of NRCMS so far has been limited to catastrophic diseases, mainly reimbursing hospitalization expenses rather than all outpatient expenses. The reality is, though outpatient expenses for each doctor’s visit are relatively low in comparison to those of a hospital stay, the cumulative expenses can be a significant amount for a household [18]. Thus, excluding outpatient expenses would definitely have had a negative impact on the effect of NRCMS. Second, the county-based NRCMS aims at financial break-even of each county pool; reimbursement schemes are developed and modified on the basis of managerial experience instead of empirical risk estimation [41], which results in low reimbursement ratios, high deductibles, low cap, and limited benefit range of reimbursement [42, 43]. Finally, although funding has increased by several fold in recent years, it remains difficult to keep up with the rapid rise of healthcare expenses. The above factors have led to the overall low levels of reimbursement and security of NRCMS [14, 44], thereby less than expected impact on reducing healthcare burden on rural residents [45].

The three issues mentioned above all point to the urgent need for an optimal reimbursement scheme that is technically sound, administratively practicable and financial sustainable. The current implementers of NRCMS do not yet know how to make the schemes financially and administratively more practicable. Nor do the implementers have clear ideas about who the target reimbursement participant should be or what the main issue is for the scheme. Lack of managerial capacity has become an important factor potentially influencing the success of rural health insurance in China, which has been corroborated by other studies [46–48]. Moreover, these problems are in accord with our conclusion from the intention survey that the primary obstacle to achieving the policy goals is to solve the technical difficulties in implementing NRCMS.

The year-2000 surveys of government departments, healthcare administrators and health professionals showed some differences from those in year 2008 on the major potential difficulties. That the technical difficulties could become primary problems in implementing NRCMS could be of various reasons. For example, the previous system (RCMS, up to 2003) with heavy reliance on township and village financing was hard to establish and maintain if the township government did not pay adequate attention or the township/village did not have sufficient own-source revenue. In contrast, the new system (NRCMS, since 2003) is co-financed by three levels of government plus participant-paid premiums. Government prioritized NCRMS with substantial outlays; besides, the central government provides extra subsidy for each participant in the poor western and interior regions [39, 40]. Thus raising the insurance premium on NRCMS was no longer as big a barrier as in 2000 and rural residents’ enthusiasm of participation improved and their confidence in NRCMS enhanced, which added to the motivation of implementing NRCMS. To further promote NRCMS, it is thus important to solve the technical difficulty in terms of actuarial funding, making appropriate reimbursement plans for implementation of the program.

By the above analysis, our findings highlight the need to establish dynamic relations according to actuarial calculations between medical need, financial risk, impoverishment from medical expenses, reimbursement ratio, maximum premiums, coverage rate, and benefit range, so as to calculate actuarially sustainable funding level and to make practicable reimbursement schedules to achieve maximum policy goals. We would recommend several key knobs for the implementers of NRCMS to improve their capacity building in the design of the reimbursement schemes: (1) Identify the financial risk through an overview of service recipients – outpatients and inpatients, patrons of county, township, and village level healthcare providers – including the probability of participants seeking healthcare service and their medical expenses. Identify households that are impoverished from health hazards, which should be the main target reimbursement group when designing the reimbursement schedules [36]. (2) Calculate the total premium level needed to eliminate the specific medical financial risk, taking into account necessary management fee, risk reserve fund, reasonable growth in medical costs, and rising medical demands, which will help to determine the funding level paid by NRCMS members [49]. (3) Conduct the preliminary evaluation of the reimbursement effect after the design or adjustment of the reimbursement schemes, including break-even of the insurance funds, pooling of financial risks, reduction of impoverishment, and improvement of income equity, which will contribute to choosing the optimal schemes.

Needless to say, this current study contains limitations. First, the pre- and post-implementation comparison that we have used in evaluating the outcome of NRCMS does not control for the endogenous effects of socioeconomic factors. Future studies should take these factors into consideration. Second, due to data limitations, our calculation has included only direct medical expenses but not indirect, non-medical costs such as food and transportation, which might have affected the accuracy of our evaluation. Third, we used three measures to evaluate the achievements of the policy goals of NRCMS in this study; whether there are better indicators to complete this issue is worth exploring. Finally, as the sampling cities are located in Eastern China, the results and conclusions of this study might reflect the trends in the well-developed areas, while extrapolations to other areas (such as the Western Region) or the entire country must be made carefully due to the differences among the regions throughout China. These limitations need to be addressed in subsequent studies.

## Conclusions

China has successfully launched NRCMS, the world’s largest health insurance program, showing the determination of the Chinese government to provide basic healthcare coverage for the country’s 900 million rural residents to solve the problems of unaffordable medical expenses and poverty due to health hazards and to promote steady rural development.

This article has offered an evaluation of the effect of China’s new medical system for rural residents, indicating that NRCMS has achieved some positive effects on alleviating health-related poverty and financial risk, and on improving income equity eroded by medical expenses. However, China’s NRCMS still faces a long uphill road in order to obtain the designed policy goals. As of now, technical difficulties in implementing NRCMS have become an eminent obstacle. Several approaches, such as identifying the financial risk and medical impoverishment, calculating actuarial funding level needed for the risk pooling and carrying on pre-appraisal for the new programs, should be introduced to the implementers of NRCMS to effectively design the reimbursement schemes, which are critical to the success of the Chinese rural health insurance system in achieving its objectives.

An examination of rural health care in China can also carry immediate and practical implications for the design and implementation of public health policies in other countries, especially developing and transitional countries.

## Abbreviations

RCMS: Rural cooperative medical system
NRCMS: New rural cooperative medical system
